# Investigation of the Active Compounds and Important Pathways of Huaiqihuang Granule for the Treatment of Immune Thrombocytopenia Using Network Pharmacology and Molecular Docking

**DOI:** 10.1155/2023/5984361

**Published:** 2023-01-10

**Authors:** Wenwen Chen, Hongtao Kan, Min Qin, Jia Yang, Wanjun Tao, XiaoYang

**Affiliations:** ^1^Department of Pharmacy, Chengdu Women's and Children's Central Hospital, School of Medicine, University of Electronic Science and Technology of China, Chengdu 610041, China; ^2^Department of Obstetrics, Chengdu Women's and Children's Central Hospital, School of Medicine, University of Electronic Science and Technology of China, Chengdu 610041, China

## Abstract

**Materials and Methods:**

Compounds of HQHG were scanned by LC-MS/MS, and the target profiles of compounds were identified based on SwissTarget Prediction. ITP target proteins were collected from various databases. Then, KEGG pathway and GO enrichment analyses were performed to explore the signaling pathways related to HQHG for ITP. The PPI and drug-herbs-compounds-targets-pathways network were constructed using Cytoscape 3.7.2. Finally, Discovery studio software was used to confirm the key targets and active compounds from HQHG.

**Results:**

A total of 187 interacting targets of 19 potentially active compounds in HQHG and 3837 ITP-related targets were collected. Then, 187 intersection targets were obtained. A total of 20 key targets including EGFR, CASP3, TNF, STAT3, and ERBB2 were identified through PPI network analysis. These targets were mainly focused on the biological processes of positive regulation of protein phosphorylation, cellular response to organonitrogen compound, and cellular response to nitrogen compound. 20 possible pathways of HQHG in the treatment of ITP were identified through KEGG enrichment. EGFR, CASP3, TNF, and STAT3 are the four most important target proteins, while adenosine, caffeic acid, ferulic acid, quercetin-3*β*-D-glucoside, rutin, scopoletin, and tianshic acid are the most important active compounds, which were validated by molecular docking simulation.

**Conclusion:**

This study demonstrated that HQHG produced relief effects against ITP by regulating multitargets and multipathways with multicompounds. And the combined data provide novel insight of drug developing for ITP.

## 1. Introduction

Primary immune thrombocytopenia (ITP) is an acquired autoimmune disease. The annual incidence rate of ITP in adults reported is (2 ~ 10)/100000, while the annual incidence rate in children is about 4 ~ 5/100000 [1, 2]. The clinical manifestations of ITP vary greatly, including asymptomatic thrombocytopenia, skin mucosal bleeding, severe visceral bleeding, and fatal intracranial hemorrhage. The main pathogenesis of ITP is the loss of immune tolerance to platelet autoantigen, resulting in abnormal activation of humoral and cellular immunity, which jointly mediates the acceleration of platelet destruction and the insufficient production of platelets by megakaryocytes [3].

Huaiqihuang Granule (HQHG), a Chinese patent medicine, is composed of Lycium barbarum, Polygonatum, and extracts of Trametes robiniophila, which could supplement the vital energy. It is applicable for children's and old people's physical weakness, such as repeated cold, dizziness, weariness, parched mouth, palpitation, hidrosis, loss of appetite, and constipation. Lycium barbarum mainly contains Lycium barbarum polysaccharide, Lycium barbarum pigment, flavonoids, alkaloids, amino acids, trace elements, and other components [4]. Polygonatum mainly contains steroidal saponins, flavonoids, phenylpropanoid, alkaloids, polysaccharides, and other components [5]. Extracts of Trametes robiniophila mainly contains Trametes robiniophila heteropolysaccharide protein, which composed of fucose, galactose, arabinose, glucose, mannose, and xylose [6, 7]. It has been reported that HQHG has the effect of regulating immune function and plays a significant role in the treatment of a variety of immune diseases, including ITP, while the specific mechanism is unclear [8–10].

**Figure 1:**
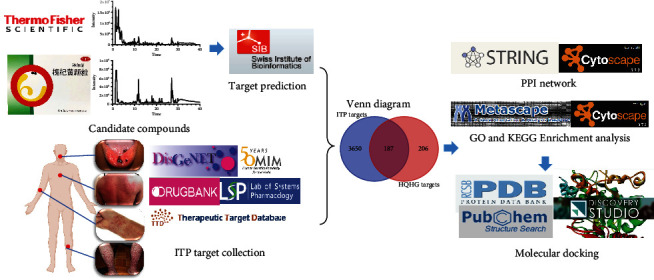
Flowchart of the analysis strategy in the study.

In this study, network pharmacology was conducted to establish the components-targets-pathways network to investigate the potential mechanisms of HQHG in the treatment of ITP [11] and verify it through molecular docking technology (Figure 1).

## 2. Materials and Methods

### 2.1. Reagents and Materials

Huaiqihuang Granules (Lot: KH23) was provided by Gaitianli Pharmaceutical Co., Ltd.

HPLC grade methanol was purchased from Fisher Scientific (USA). Water was purified using a ULUPURE water system (UPT-II-1, China). Other chemicals were of analytical grade.

### 2.2. Identification of Active Compounds in HQHG

HQHG (5 g) was mixed with methanol (10 ml), ultrasonic for 15 min, and the supernatant was filtrated and collected, then centrifuged at 3000 rpm for 5 min. The supernatant was filtered through a 0.22 *μ*m microporous membrane. The active compounds of HQHG extracts were identified by Q Exactive Orbitrap LC-MS/MS (Thermo Fisher Scientific). The XBridgeBEHC18 (2.1 × 100 mm, i.d.; 2.5 *μ*m, waters) was applied for sample separation at 35°C. Methanol (A) and 0.1% formic acid-water (B) (95 : 5) were used as mobile phase, and the solvent gradient was set as follows: 5% B, 0 min; 95% B, 40.0 min. Detection wavelength was set at 280 nm. Nitrogen was applied as auxiliary gas and sheath gas at a flow rate of 12 l/min. The mass determination was carried out based on positive and negative scanning mode with the m/z of 100-1000.

### 2.3. Acquisition of Corresponding Targets of Active Compounds in HQHG

All the chemical structures of the identified active compounds were imported into SwissTarget Prediction (http://www.swisstargetprediction.ch/) to predict the targets. The probability of targets greater than 0.6 was collected. Targets of these compounds were collected through high-throughput screening and reverse docking. The corresponding targets of Trametes robiniophila were collected from literatures and merged with other targets.

### 2.4. Corresponding Targets of ITP Collection

Keywords such as “immune thrombocytopenia,” “thrombocytopenia immunologic,” or “idiopathic thrombocytopenic purpura” were used to search the known targets related to ITP from some databases, including DisGeNET (https://www.disgenet.org/) [12], TCMSP (http://tcmspw.com/index.Php) [13], DrugBank (https://go.drugbank.com/) [14], Therapeutic Target (https://db.idrblab.net/ttd/) [15], GeneCards (https://www.genecards.org) [16], and OMIM (https://omim.org/) [17]. The chemical components without corresponding targets were removed, and the repeated targets were deleted. The intersection targets of active components in HQHG and immune thrombocytopenia were acquired by Venn diagram

Protein-protein interaction (PPI) network

The intersection targets were imported into the STRING database (https://string-db.org/) to obtain PPI network, with the species of human and a highest confidence of 0.9 [18]. The PPI network was visualized by Cytoscape software (version 3.7.2) [19]. The key targets were selected depend on degree for molecular docking.

### 2.5. GO and KEGG Pathway Enrichment Analyses

The Gene Ontology (GO) and Kyoto Encyclopedia of Genes and Genomes (KEGG) enrichment were performed on the Database for Metascape (https://metascape.org) [20]. GO functionally annotates key genes into 3 main terms, i.e., cellular components (CCs), molecular functions (MFs), and biological processes (BPs). KEGG enrichment analysis unveils the possible biological process with key targets. The terms of CCs, MFs, and BPs that were significantly enriched (*P* value 0.05, minimum count of 3, and enrichment factor of >1.5) were displayed. The bubble chart of GO and KEGG enrichment analyses was performed on the bioinformatics platform (http://www.bioinformatics.com.cn/).

### 2.6. Construction of Drug-Components-Disease-Targets-Pathways Network

A network of drug-components-disease-targets-pathways was constructed to characterize the therapeutic mechanisms of HQHG for ITP. The nodes with different colors and shapes in the network represent the drug, components, disease, targets, or disease-related pathways, respectively, and the “edge” between the nodes shows the association.

### 2.7. Molecular Docking Simulation

According to the KEGG path analysis results, the related targets were screened from the rcsbpdb database (http://www.rcsb.org/) [21]. Compounds were filtered by Lipinski's and Veber's rules with Discovery studio software (version 4.5.0, Biovea Inc., Omaha, NE, USA) [22] and prepared by “Prepare Ligands” module, while pretreat protein crystals were prepared by the “Prepare Protein” module. Subsequently, molecular docking was performed in “LibDock” module, and LibDockScore was required to evaluate affinity of the target proteins and active components. The LibDockScore of the target protein and its corresponding prototype ligand was viewed as the threshold, and the components with higher scores were regarded as the active compounds of HQHG.

## 3. Results

### 3.1. The Active Components in HQHG

**Figure 2:**
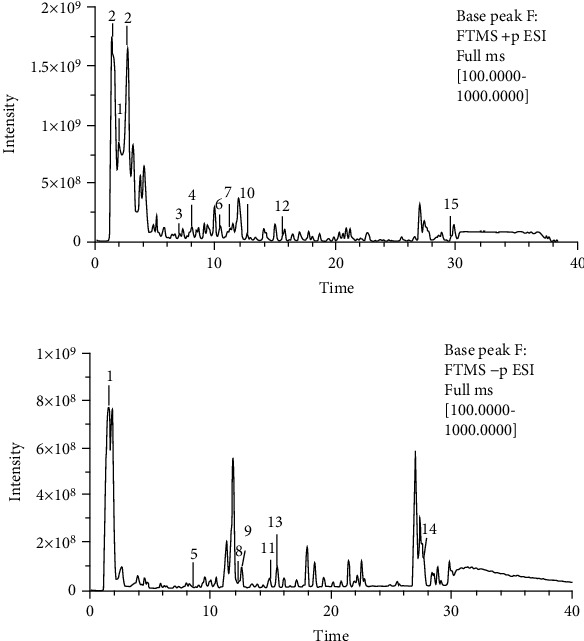
Chromatogram of HQHG by Q Exactive Orbitrap LC-MS/MS on positive-ion polarity mode (a) and negative-ion polarity mode (b).

**Figure 3:**
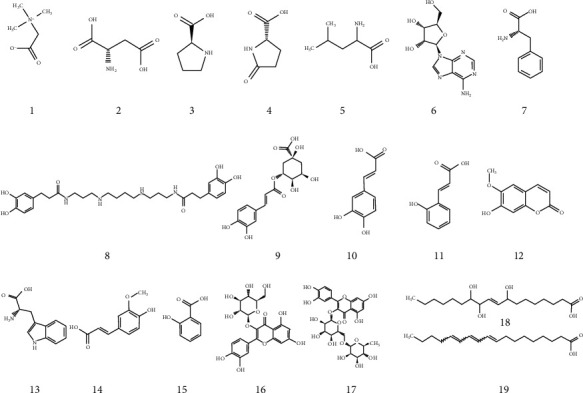
Compounds detected in HQHG by Q Exactive Orbitrap LC-MS/MS.

As shown in Figure 2, the total ion chromatogram (TIC) was acquired by the Q Exactive Orbitrap LC-MS/MS system, with the positive mode (A) and negative mode (B). More than 40 major compounds were eluted from the HQHG within 40 min. A total of 19 compounds in Lycium barbarum and Polygonatum were finally identified by comparing with the known chemical constituents in the reported literature data and deduced according to their mass spectrometry and fragment ion characteristics, as shown in Table 1 and Figure 3.

### 3.2. Components in HQHG and Candidate Targets Associated with ITP

**Figure 4:**
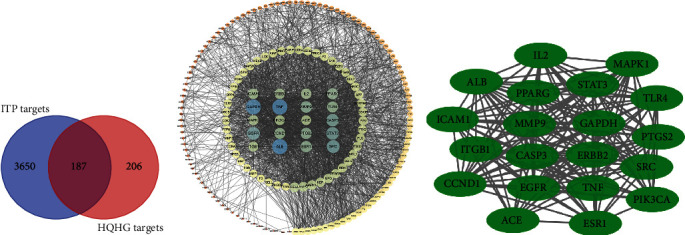
The representative pictures of Venn diagram and PPI network. (a) 187 intersection targets; (b) a complete protein-protein network; (c) key target network with 20 nodes and 168 edges.

As shown in Figure 4(a), a total of 393 corresponding targets of compounds were acquired from SwissTarget Prediction after repeated targets deleted. A total of 3837 ITP targets were retrieved and integrated from the databases of DisGeNET, TCMSP, DrugBank, GeneCards, TTD, and OMIM. A total of 187 intersection targets were obtained by combining the corresponding targets of the active component of HQHG with the related targets of ITP.

### 3.3. PPI Network Analysis

The intersection targets were input into the STRING database, and the PPI network diagram were drawn by the Cytoscape software, as shown in Figure 4(b). There are 187 nodes and 4490 edges, and the average degree of nodes is 24. The top targets, EGFR, CASP3, SRC, TNF, MMP9, STAT3, and ERBB2, were selected as the core targets for molecular docking with the core compound of HQHG for ITP treatment. The target with the degree value greater than 2 times the median was regarded as the key targets (Figure 4(c)). Ultimately, 20 key targets, PIK3CA, MAPK1, IL2, MMP9, ITGB1, TLR4, PPARG, ICAM1, ACE, ESR1, TNF, SRC, PTGS2, CASP3, ALB, EGFR, ERBB2, STAT3, GAPDH, and CCND1, were collected for pathway enrichment analysis (Table 2).

### 3.4. GO and KEGG Enrichment Analyses

**Figure 5:**
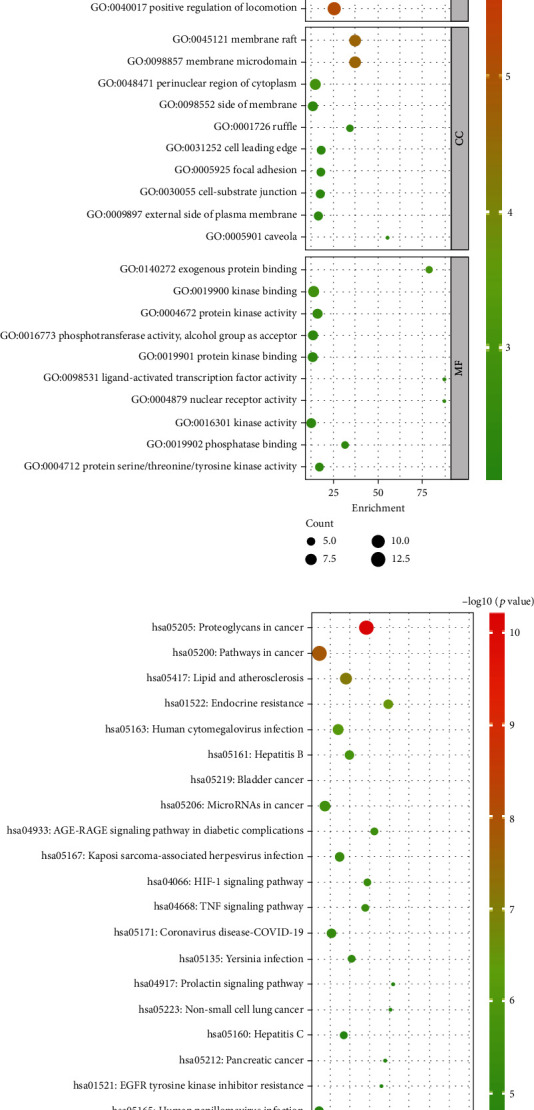
GO and KEGG enrichment analyses based on 20 key targets. (a) Top 10 significantly enrichment on BPs, CCs, and MFs. (b) Top 20 significantly enriched pathways.

The top 10 significantly enriched BPs, CCs, and MFs' terms were shown in Figure 5(a) (*p* < 0.01). The size of the dot in bubble chart indicates the number of target genes in the corresponding function pathway, and the enrichment expresses the ratio of the number of target genes belonging to all the annotated genes located in the pathway. For BPs, the top 3 significant enrichment with the key targets were GO:0001934 positive regulation of protein phosphorylation, GO:0071417 cellular response to organonitrogen compound, and GO:1901699 cellular response to nitrogen compound. For CCs, the top 3 significant enrichment were GO:0045121 membrane raft, GO:0098857 membrane microdomain, and GO:0048471 perinuclear region of cytoplasm, while GO:0140272 exogenous protein binding, GO:0019900 kinase binding, and GO:0004672 protein kinase activity were the top 3 significant enrichments in MFs.

The KEGG enrichment showed how HQHG acts on the pathway, thereby playing a therapeutic role in ITP. As shown in Figure 5(b), top 20 significant signaling pathways (*p* < 0.01) were picked out for further analysis based on the 20 key target genes, including hsa05205 Proteoglycans in cancer, hsa05200 Pathways in cancer, and hsa05417 Lipid and atherosclerosis as the top ones.

### 3.5. Drug-Components-Targets-Pathways Network

**Figure 6:**
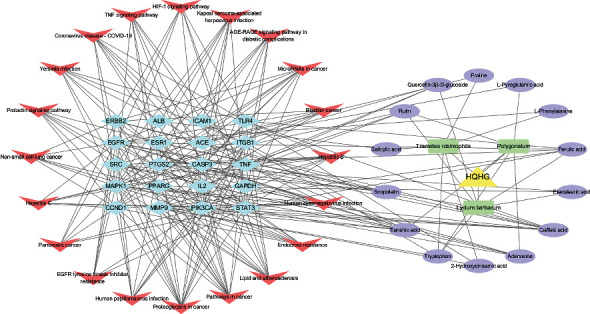
The drug-herbs-compounds-targets-pathways network. The yellow triangle nodes indicate the HQHG, the green round rectangle nodes indicate herbs, the purple ellipse nodes indicate compounds in HQHG, the blue diamond nodes indicate the top 20 targets, and the red v shape nodes indicate the top 20 pathways. Lines represent the interactions between the nodes.

The drug-components-targets-pathways network was shown in Figure 6, which included 58 nodes (1 drug, 3 herbs, 14 compounds, 20 targets, and 20 pathways) and 224 edges. According to the network analysis, multiple compounds of HQHG acts on at least one target genes, and caffeic acid was regarded as the most effective compound that interacts with 8 target genes. Besides, most of target genes were regulated by at least 2 active compounds, and at least 7 genes potentially involved in each pathway related to ITP. This network analysis indicated the characteristics of multiple components and multiple targets of HQHG in the treatment of ITP.

### 3.6. Molecular Docking Results and Analysis

**Figure 7:**
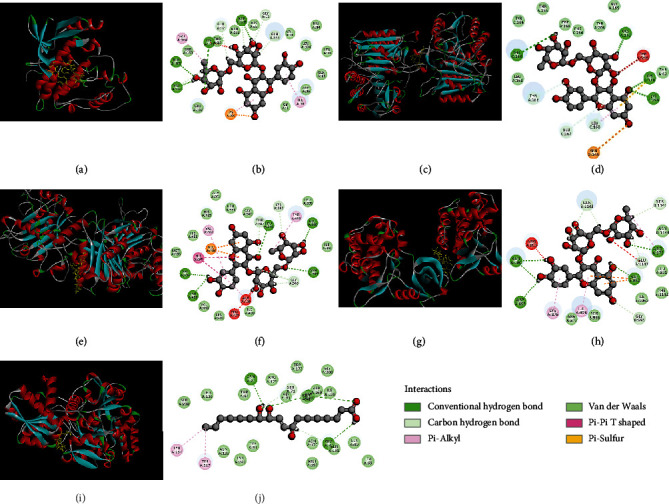
The represented results for the action mode of active compounds with 5 targets protein using molecular docking. (a, b) Action mode of rutin with target EGFR (PDB ID: 4RJ3): rutin has conventional hydrogen bonding interactions with ASP145, LYS129, THR165, and VAL163 of EGRF; carbon hydrogen bonding interactions with GLY13, GLN131, and GLU12; Pi-alkyl interactions with VAL164 and ILE10; Pi-Sulfur interaction with LYS88. (c, d) Action mode of rutin with CASP3 (PDB ID: 3DEK): rutin has conventional hydrogen bonding interactions with THR166, OSC163, CYS170, and LYS259 of CASP3; carbon hydrogen bonding interactions with THR166, LEU168, and GLU167; Pi-Sulfur interaction with ARG144. (e, f) Action mode of rutin with TNF (PDB ID: 2I47): rutin has conventional hydrogen bonding interactions with TYR436, VAL434, ASN389, GLU398, and LEU348 of TNF; carbon hydrogen bonding interactions with THR347, GLY346, and LYS392; Pi-Pi T shape interaction with HIS405; Pi-alkyl interactions with VAL402 and TYR390; Pi-Sulfur interaction with GLU406. (g, h) Action mode of rutin with STAT3 (PDB ID: 5E1E): rutin has conventional hydrogen bonding interactions with ASN900, ASP895, ASP992, and ARG879 of STAT3; carbon hydrogen bonding interactions with GLN1143, SER1141, GLU1147t, and GLY995; Pi-alkyl interactions with LYS876 and ILE878. (i, j) Action mode of tianshic acid with target ERBB2 (PDB ID: 3PPO): tianshic acid has conventional hydrogen bonding interactions with ASN72, GLY70, and GLN39 of ERBB2; Pi-alkyl interactions with TYR137 and TYR217.

A total of 5 target genes in “pathways in cancer” which showed strong interactions with other targets, namely, EGFR, CASP3, TNF, STAT3, and ERBB2, were selected to binding with active compounds in HQHG, including caffeic acid, tianshic acid, scopoletin, ferulic acid, adenosine, rutin, and quercetin-3*β*-D-glucoside. LibDockScore of the testing compounds was acquired to indicate the binding affinities with each target (Table 3). All the testing compounds had strong interactions than the prototype ligands or similar effects to the ligands, with EGFR, CASP3, TNF, STAT3, and ERBB2. Interestingly, rutin showed good binding affinities with all the tested targets, except ERBB2. In addition, tianshic acid also showed a strong interaction with all targets, while quercetin-3*β*-D-glucoside showed good binding affinities with EGFR, CASP3, TNF, and STAT3. These findings revealed that rutin, tianshic acid, and quercetin-3*β*-D-glucoside could be forecasted as the active compounds of HQHG for ITP, while EGFR, CASP3, TNF, and STAT3 were the major targets for reaching this effect. The representative molecular docking results of the major targets and active compounds of HQHG were exhibited in Figure 7.

## 4. Discussion

Primary immune thrombocytopenia is an autoimmune bleeding disorder, and the risk of fatal hemorrhage of ITP is around 1.6–3.9% per year [31]. The first-line treatment of ITP mainly includes adrenal glucocorticoid and human immunoglobulin, while the second-line treatment mainly includes thrombopoietin and rituximab or splenectomy [31]. Due to the drug resistance, adverse reactions, and high treatment costs of these drugs, it is necessary to find therapeutic drugs with relatively low toxicity and cost, such as traditional Chinese medicine.

A total of 20 key genes were screened in this study as the most important ones that contribute to ITP treatment of HQHG. These potential target genes were placed in the Metascape for KEGG pathway analysis, and 20 significant pathways that may be regulated by HQHG in the treatment of ITP were identified, including pathways in cancer, lipid and atherosclerosis, endocrine resistance, human cytomegalovirus infection, hepatitis B, microRNAs in cancer, AGE-RAGE signaling pathway in diabetic complications, HIF-1 signaling pathway, and TNF signaling pathway. Interestingly, the relationships between some pathways and ITP were also confirmed in other studies [32–35]. It has been reported that impaired megakaryocyte maturation and exaggerated platelet destruction are critical in the pathogenesis of immune thrombocytopenia. Molecular mimicry is mainly involved in secondary acute ITP, accounting for 70% of neonatal cytomegalovirus (CMV). Indeed, the platelet count drops as a result of the increase in macrophage phagocytosis secondary to stimulation by IFN-*γ* elicited by viruses [36]. Human cytomegalovirus (HCMV) infection pathway was proved that myeloid HCMV infection is a specific factor in children's ITP, and the patients of ITP with myeloid HCMV infection had a tendency for exacerbation, refractoriness, and chronic advance, which also be observed in mice [37, 38]. Pathways in cancer were reported to be the most potentially associated with ITP from Chinese Han population [39]. HIF-1*α* signaling pathway was reported that decreased HIF-1*α* may contribute to impaired megakaryopoiesis in ITP and may provide a potential therapy for ITP patients [33]. TNF-*α* blockade decreased antibody-mediated platelet destruction and may be a promising therapeutic strategy for the management of ITP [32]. Anti-TNF-*α* therapy reduced the number of nonclassical monocytes and M1 macrophages, ameliorated the retention of platelets in spleen and liver, and increased the platelet count of ITP mice [32]. To activate the Janus Kinase/Signal Transducer and Activator of Transcription (JAK/STAT) pathway, megakaryocyte proliferation and platelet production could be activated. Therefore, we chose EGFR, CASP3, TNF, STAT3, and ERBB2 target genes in these pathways to further molecular docking based on references and the PPI results of this study [40–42].

In this study, rutin showed the highest LibDockScores and forms many close interactions including conventional hydrogen bonds, Pi-Sulfur, Pi-alkyl, and carbon hydrogen bond to the residues of EGFR, CASP3, TNF, and STAT3, which indicated that rutin in HQHG plays an important role in the treatment of ITP. Rutin has been exploited for its potential role in inhibiting cancer cell growth and metastasis. It is proved that rutin could modulate STAT signaling via the repression of SRC phosphorylation and block the translocation of STAT to the nucleus [43] and further block the activation of TWIST1, MMPs, and VEGF via the inhibition of STAT3 phosphorylation [44]. Nevertheless, there is no research of rutin on the potential role in ITP treatment, and this study may be helpful for the development of new therapeutic uses of rutin. Another important active compound, caffeic acid, was also noteworthy. Caffeic acid, one of the most common phenolic acids, exhibited compact relationships with 8 key target genes, which indicates its importance in ITP treatment of HQHG. Caffeic acid was reported with a wide range of pharmacological properties, such as anti-inflammation, antioxidant, anticancer bioactivities, immunomodulatory, and neuroprotective activities [45]. Caffeic acid could downregulate IL-6, IL-1*β*, and NF-*κ*B in the inflammatory response [46], drastically block STAT3 action, and thus down-trigger HIF-1*α* action [47]. A clinic trial in China was verified that caffeic acid was effective in patients with ITP [48]. Other active components, like adenosine, a purinergic signaling molecule, showed good binding affinities with EGFR, TNF, STAT3, and ERBB2 than prototype ligands and were a well-known actor of the immune system and the inflammatory response both in physiologic and pathologic conditions [49, 50]. It is hypothesized that HQHG blocks TNF-*α* signaling pathway, resulting in remarkable attenuation of antibody-mediated platelet destruction [32].

This study investigated the intricate mechanism of HQHG treatment for ITP by utilizing an integrated network pharmacological and pharmacokinetics strategy. These novel findings provide a rational pharmacological basis and support for treating ITP and may help to expand the use of this formula in the treatment of ITP. However, several limitations should be noted in this study. Firstly, large molecular compounds such as polysaccharides and proteins in HQHG were not included in this study, and it still needs to focus on further research. Secondly, the underlying mechanism of HQHG against ITP involves multiple pharmacological actions, and more research is needed to reveal complex synergistic mechanisms by combining high-throughput detection technologies such as transcriptome, proteome, and metabolome. Moreover, the contribution of the active ingredients to efficacy and the synergistic effect among the active ingredients were not explained in our research, and we will conduct research in this area in the future to more fully interpret the modern theory of TCM efficacy.

## 5. Conclusion

Collectively, 19 active compounds were identified from HQHG, and 187 targets of active compounds in addition to 3837 ITP-related targets were collected. A total of 20 key targets were screened out in PPI analysis, and signaling pathways including pathways in cancer, lipid and atherosclerosis, endocrine resistance, human cytomegalovirus infection, hepatitis B, microRNAs in cancer, AGE-RAGE signaling pathway in diabetic complications, HIF-1 signaling pathway, and TNF-*α* signaling pathway were observed to play an important role in the mechanism of HQHG for ITP treatment. In addition, EGFR, CASP3, TNF, and STAT3 were speculated to be the most important target proteins. Network pharmacology results showed a close relationship between the TNF-*α* pathway and ITP, while HQHG is capable of blocking the TNF-*α* pathway. This study provided novel indications for further mechanism research of HQHG in the treatment of ITP. However, more research is necessary to make the results available.

## Figures and Tables

**Table 1:** Compositions and their product ions in HQHG.

No	Name	RT (min)	Formula	Ion source model	Observed MS_1_ (m/z)	MS_2_ (m/z)	Source	Reference
1	Betaine	1.35	C_5_H_11_NO_2_	[M-H]+	118.0865	59.07368, 58.06586	A	[23]
2	L-aspartic acid	1.49	C_4_H_7_NO_4_	[M-H]-	132.0294	115.00281, 88.03943, 71.01283	B	[24]
3	Proline	1.62	C_5_H_9_NO_2_	[M-H]+	116.0711	70.06579	B	[24]
4	L-pyroglutamic acid	1.91	C_5_H_7_NO_3_	[M-H]+	130.0502	130.05005, 84.04498	C	[25]
5	DL-leucine	2.04	C_6_H_13_NO_2_	[M-H]-	130.0867	88.03941, 86.03180	B	[24]
6	Adenosine	2.48	C_10_H_13_N_5_O_4_	[M-H]+	268.1043	136.06187, 119.03543	C	[25]
7	L-phenylalanine	3.74	C_9_H_11_NO_2_	[M-H]-	164.0712	147.04443, 120.04465, 72.00810	B	[24]
8	Kukoamine A	6.86	C_28_H_42_N_4_O_6_	[M-H]+	531.3178	352.60428, 293.18582, 222.11252, 165.05467, 123.04430	A	[26]
9	Chlorogenic acid	8.01	C_16_H_18_O_9_	[M-H]+	355.1028	193.04965, 163.03859, 133.02855	A	[4]
10	Caffeic acid	8.56	C_9_H_8_O_4_	[M-H]-	179.0346	135.04431, 134.03650, 89.02342	A	[27]
11	2-Hydroxycinnamic acid	11.17	C_9_H_8_O_3_	[M-H]+	165.0549	147.04402, 119.04935, 91.05470	C	[25]
12	Scopoletin	11.80	C_10_H_8_O_4_	[M-H]+	193.0499	178.02615, 137.05978, 133.02849	A	[23]
13	Tryptophan	12.58	C_11_H_12_N_2_O_2_	[M-H]-	203.0824	159.09200, 142.06537, 116.04964	A/B/C	[24, 27–30]
14	Ferulic acid	12.63	C_10_H_10_O_4_	[M-H]+	195.0657	177.05472, 149.05984, 145.02849	A	[26, 27]
15	Salicylic acid	15.07	C_7_H_6_O_3_	[M-H]-	137.0238	93.03357	C	[28]
16	Quercetin-3*β*-D-glucoside	15.54	C_21_H_20_O_12_	[M-H]+	465.1032	303.05008, 257.04443, 229.04915, 85.02902	A	[27]
17	Rutin	15.54	C_27_H_30_O_16_	[M-H]-	609.1462	300.02756, 271.02493, 255.02986	A/C	[27, 28]
18	Tianshic acid	27.66	C_18_H_34_O_5_	[M-H]-	329.2333	171.10193, 139.11200, 127.11195	C	[28]
19	Eleostearic acid	29.46	C_18_H_30_O_2_	[M-H]+	279.2318	149.02348, 123.11689, 109.10156, 95.08602	A	[26]

A: Lycium barbarum; B: Trametes robiniophila; C: Polygonatum.

**Table 2:** The key targets of HQHG for ITP treatment and the topological parameters.

Gene name	Target name	UniProt ID	Target class	BetweennessCentrality	ClosenessCentrality	Degree	TopologicalCoefficient
ALB	Serum albumin	P02768	Secreted protein	0.116336	0.69924812	111	0.238997
TNF	TNF-alpha	P01375	Secreted protein	0.087174	0.69662921	110	0.248704
GAPDH	Glyceraldehyde-3-phosphate dehydrogenase liver	P04406	Oxidoreductase	0.103707	0.69144981	107	0.240194
SRC	Tyrosine-protein kinase SRC	P12931	Kinase	0.042761	0.62207358	83	0.237859
EGFR	Epidermal growth factor receptor erbB1	P00533	Kinase	0.049119	0.62207358	82	0.254689
STAT3	Signal transducer and activator of transcription 3	P40763	Transcription factor	0.030602	0.61589404	81	0.232068
CASP3	Caspase-3	P42574	Protease	0.027922	0.61386139	78	0.220249
MMP9	Matrix metalloproteinase 9	P14780	Protease	0.015639	0.57943925	68	0.249442
ESR1	Estrogen receptor alpha	P03372	Nuclear receptor	0.025108	0.58490566	65	0.226744
PTGS2	Cyclooxygenase-2	P35354	Oxidoreductase	0.019666	0.5830721	65	0.219843
PPARG	Peroxisome proliferator-activated receptor gamma	P37231	Nuclear receptor	0.024164	0.5830721	63	0.180814
TLR4	Toll-like receptor 4 (by homology)	O00206	Toll-like and Il-1 receptors	0.011561	0.56534954	62	0.209176
CCND1	Carbonyl reductase [NADPH] 1	P16152	Enzyme	0.017068	0.57230769	61	0.231736
IL2	Interleukin-2	P60568	Secreted protein	0.012506	0.56363636	56	0.218021
ERBB2	Receptor protein-tyrosine kinase erbB-2	P04626	Kinase	0.009878	0.56193353	56	0.174705
ICAM1	Isoleucyl-tRNA synthetase	P41252	Enzyme	0.006515	0.55855856	55	0.206215
MAPK1	MAP kinase ERK2	P28482	Kinase	0.008731	0.55522388	51	0.2468
PIK3CA	PI3-kinase p110-alpha subunit	P42336	Enzyme	0.008804	0.54069767	49	0.215699
ITGB1	Intercellular adhesion molecule (ICAM-1), integrin alpha-L/beta-2	P20701 P05362 P05107	Membrane receptor	0.006189	0.55192878	49	0.180651
ACE	Angiotensin-converting enzyme	P12821	Protease	0.015263	0.54867257	48	0.232635

**Table 3:** The LibDockScore of core targets and their interacting compounds.

	EGFR	CASP3	TNF	STAT3	ERBB2
PDB ID	4RJ3	3DEK	2I47	5E1E	3PPO
Ligand	36.2757	127.085	150.675	50.1198	79.9372
Adenosine	105.345	78.4298	126.119	87.4359	123.208
Caffeic acid	72.1329	56.7253	99.0207	65.7432	89.7552
Ferulic acid	75.7277	57.8825	95.146	67.7231	92.0158
Quercetin-3*β*-D-glucoside	135.829	117.332	164.278	127.756	—
Rutin	152.242	127.796	180.199	144.249	—
Scopoletin	74.2542	60.6044	100.557	66.1219	88.9612
Tianshic acid	129.282	105.871	143.458	110.537	142.933

## Data Availability

All data are available in the manuscript, and they are exhibited in figures and tables.
